# Can we quantify harm in general practice records? An assessment of precision and power using computer simulation

**DOI:** 10.1186/1471-2288-13-39

**Published:** 2013-03-13

**Authors:** Carl de Wet, Paul Johnson, Catherine O’Donnell, Paul Bowie

**Affiliations:** 1 NHS Education for Scotland, 2 Central Quay, Glasgow, G3 8BW, UK; 2 Robertson Centre for Biostatistics, Institute of Health and Wellbeing, University of Glasgow, Glasgow, UK; 3 General Practice & Primary Care, Institute of Health & Wellbeing, College of Medical, Veterinary and Life Science, University of Glasgow, Glasgow, UK

**Keywords:** Patient safety, General practice, Primary care, Trigger tool, Clinical record review, Precision, Power, Harm

## Abstract

**Background:**

Estimating harm rates for specific patient populations and detecting significant changes in them over time are essential if patient safety in general practice is to be improved. Clinical record review (CRR) is arguably the most suitable method for these purposes, but the optimal values and combinations of its parameters (such as numbers of records and practices) remain unknown. Our aims were to: 1. Determine and quantify CRR parameters; 2. Assess the precision and power of feasible CRR scenarios; and 3. Quantify the minimum requirements for adequate precision and acceptable power.

**Method:**

We explored precision and power of CRR scenarios using Monte Carlo simulation. A range of parameter values were combined in 864 different CRR scenarios, with 1000 random data sets generated for each, and harm rates were estimated and tested for change over time by fitting a generalised linear model with a Poisson response.

**Results:**

CRR scenarios with ≥100 detected harm incidents had harm rate estimates with acceptable precision. Harm reductions of 20% or ≥50% were detected with adequate power by those CRR scenarios with at least 100 and 500 harm incidents respectively. The number of detected harm incidents was dependent on the baseline harm rate multiplied by: the period of time reviewed in each record; number of records reviewed per practice; number of practices who reviewed records; and the number of times each record was reviewed.

**Conclusion:**

We developed a simple formula to calculate the minimum values of CRR parameters required to achieve adequate precision and acceptable power when monitoring harm rates. Our findings have practical implications for health care decision-makers, leaders and researchers aiming to measure and reduce harm at regional or national level.

## Background

It is now generally accepted that a significant minority of patients may suffer preventable harm as a result of their interaction with health care [1]. In response, improving patient safety has become a national priority of most modern health care systems, including the National Health Service (NHS) in the United Kingdom (UK). Initial efforts were mainly directed at acute hospitals, but the improvement and research focus are now being widened to include other care settings, including primary care and general medical practice [2,3].

To begin to consider how to measure and improve the safety of general practice, at least two important challenges have to be considered. Although a number of explicit patient safety risks have been identified, the incidence of harm in primary care has not yet been quantified reliably [4]. This is essential to understand the scale of the safety problem and to inform the design and implementation of improvement initiatives. The second challenge is to evaluate the impact of these interventions through serial monitoring to determine if improvement efforts are beneficial and leading to safer care or to otherwise adapt them to ensure they are.

The review of clinical records may offer a consistent and widely applicable approach to solving both of these problems. The findings of landmark studies utilizing this approach have shaped our understanding of the scale of the problem in secondary care settings worldwide and this evidence was the primer for the development and implementation of national policies to make patient care safer [5,6]. Clinical record review (CRR) is a well-established approach to detecting and quantifying sub-optimal care issues [7,8]. CRR allows estimation of harm rates for specific patient populations at given points in time and, if repeated, allows comparisons to detect significant changes across time. Other methods such as incident reporting systems and analyzing complaints are methodologically limited by comparison regarding this specific aspect because they rely on self-report data or typically focus on only a small sub-set of the patient population [9-12].

The CRR method is flexible with no single ‘correct’ adaptation. In recent years the Institute for Healthcare Improvement (IHI) has popularized the ‘global trigger tool’ as a means for frontline clinicians to estimate harm rates using this rapid, focused and structured approach to record review [13]. Their rationale for the trigger tool method is ‘…the ability to quantify [harm] accurately with relatively small samples [of medical records] and to follow changes [in harm rates] longitudinally over time… [12]’.

Specific trigger tools are now routinely used in many acute hospital settings worldwide [13-16] and have been piloted in other settings including primary care [17-20] to test their feasibility in measuring rates of harm. However, the reliability (and therefore potential usefulness) of these measures is dependent on a large number of CRR parameters including: (i) the quality of the clinical records; (ii) individual reviewer factors (inter-rater reliability, quality and intensity of previous training and experience, and if the reviewer is internal or external to the practice and if one or more individuals review each record); (iii) specific characteristics of the review process (number of months or patient encounters reviewed in each record, how many records are reviewed and how often reviews are conducted) and (iv) the ‘frailty’ of the patient population whose records are reviewed (inter-patient and inter-practice variation, which reflects the likelihood of patients experiencing harm) [13-20].

It remains unclear which combinations of these parameters represent suitably robust and rigorous review methods to ensure their harm rate estimates are sufficiently precise and have adequate statistical power to differentiate actual changes from random variation. Unfortunately this crucial point is often overlooked by researchers and policy makers who appear to accept any harm rate estimate or reduction at face value. Therefore, while the record review method and its various adaptations may potentially be of great use, it is essential to first resolve this methodological and statistical challenge. The aims of this study were therefore to:

1. Describe the parameters which constitute a CRR and select a range of values representative of and feasible in a general practice setting;

2. Assess the levels of precision and power of harm rate monitoring yielded by the different CRR scenarios;

3. Determine and describe the minimum requirements which ensure CRR harm rate estimates have adequate precision and acceptable power.

## Method

### Study design

We chose Monte Carlo simulation to examine the levels of precision and power of harm rate estimates as measured by different general practice CRR scenarios. Monte Carlo simulation is often used to model non-deterministic systems with substantial inherent uncertainty, which makes it ideal for the general practice setting. The key advantage of this approach is that it can be used to investigate multiple complex ‘real-life’ scenarios not covered by conventional analytical approaches. Details of how harm incidents events were simulated are given in the Statistical Modeling section below.

We identified the parameters that constitute a CRR, devised explicit assumptions about the range of parameter values that may be feasible in general practice and defined when CRR harm rate estimates would be considered to have acceptable precision and adequate power.

### Precision and power of harm rate estimates

CRR harm rate estimates can be used in two different ways. Firstly, to quantify the incidence of harm in defined patient populations at given points in time (e.g. the number of unintentional harm incidents detected in the clinical records of a population of patients with diabetes mellitus). The findings are expressed as a rate such as ‘number of harm incidents per 100 patients per year’. Secondly, harm rate estimates at different points in time can be compared to detect increases or reductions, with observed changes expressed as percentages.

#### Precision

The precision of a harm rate estimate is its repeatability, that is, the degree to which it is subject to random sampling error. We used ‘estimation error’ as a proxy for precision (low estimation error implying high precision) because it is easier to quantify and interpret. Estimation error was defined as the distance from the 95% confidence limits to the estimate (expressed as a percentage). For example, a harm rate of 10 incidents/100patients/year with a 95% confidence interval (CI) of 8–12 could also be expressed as 10 ±2. Expressing ±2 as a percentage of 10 gives an estimation error of ±20%.

A high level of precision (indicative of low estimation error) is a desirable property of any estimate, but may require substantial resources (e.g. more time, multiple reviewers and larger numbers of patient records). The converse is true for estimates with low levels of precision (high estimation errors). We pragmatically defined ‘acceptable precision’ of a harm rate estimate as an estimation error less than ±25%. In other words, any CRR harm rate estimate within ±25% of the estimated harm rate.

#### Power

In statistical terms, power is defined as the probability that a test will correctly reject the null hypothesis of no change when it is false. In the context of our study, power gauges how likely specific CRR scenarios were to detect real changes in harm rates over the simulated 12-month period. We defined ≥80% power as adequate as per convention.

### CRR parameters

We identified parameters and chose a realistic range of fixed and variable values which may affect the precision and power associated with CRR results (Table 1).

**Table 1:** Simulated clinical record review (CRR) parameters and parameter values and their effect on the precision and power of harm rate estimates

**Parameters**	**Selected parameter values**	**Parameter affects precision**	**Parameter affects power**
**Parameters with variable values**			
Number of general practices conducting CRR	1, 10, 20, 50, 100, 150, 200, 250, 300	√	√
Number of unique patient records reviewed by each practice at a given point in time	20, 25, 50, 100, 150, 200	√	√
The real harm rate in the sampled patient population, expressed as incidents per 100 patients per year	2, 5, 10, 20	√	√
The actual reduction in the real harm rate over 12 months	20%, 50%		√
Inter-patient variation in harm susceptibility, expressed as a median rate ratio (MRR)	1.2, 2	√	√
**Parameters with fixed values**			
Inter-practice variation in harm susceptibility, expressed as a MRR	1.2	√	√
Period of time reviewer reviewed in each record (calendar months)	3	√	√
Period of time over which changes in harm rates are examined (months)	12		√
Number of reviews during the simulated 12-month period	2		√
Reviews at different time points are conducted on the same or different samples of patient records	Same		√

The different parameters and parameter values can potentially be combined in 864 uniquely different CRR scenarios.

#### Fixed parameter values

We identified a number of fixed parameter values, based on previous experience of feasibility [21]; these were identical in every CRR scenario. The fixed parameters were: (i) the same internal clinical reviewer conducting reviews at (ii) two time points (the beginning and end) of a (iii) 12-month time interval using (iv) the same sample of patient records and (v) reviewing the preceding three calendar months in each unique patient record at each time point.

#### Variable parameter values

Our assumption of the real harm rates in different patient populations was based on previous research and covered a tenfold range from two to 20 incidents per 100 patients per year [1,17,18]. The lower harm rates represent a sample of patients from the whole practice population and the higher rates reflect more harm-prone samples such as elderly populations with multiple morbidities.

The number of simulated practices participating in CRR ranged from a single practice to a maximum of 300. This choice was informed by the feasibility of sample size and that there are fewer general practices than this in any given NHS Scotland regional Health Board [22]. At both time points, an internal clinical reviewer in each simulated practice reviewed a number of records which ranged from 20 to 200. Twenty records is feasible for a single internal clinician reviewer [20,23], while reviewing 200 records per time point is entirely possible using external reviewers but will clearly require greater investment in terms of time, commitment, effort and funding. Harm rate reductions of 20% (relatively small) or 50% (relatively large) were chosen for the specified 12- month period.

Patients differ in their risk of suffering harm and general practices differ in their risk of potentially causing iatrogenic harm. For example, elderly patients with multiple co-morbidities and many repeat prescription items are more likely to suffer harm than healthy young adults who infrequently attend general practice. Additionally, there is variation between practices in the frailty of patient populations, and in adherence to safety protocols, effectiveness of team working and communication, safety culture perceptions and the content quality of available records in practices [24]. We accounted for these risk variations by introducing two additional parameters: inter-patient variation and inter-practice variation (inter-practice variation can also be viewed as correlation between patients within practices). These parameters are discussed below.

#### Inter-patient and inter-practice variation

We quantified inter-patient and inter-practice variation using median rate ratios (MRR) [25]. The MRR is the expected harm rate ratio between two randomly sampled patients or practices with otherwise identical characteristics. Because the MRR is always expressed as the ratio of the higher to the lower risk, it is always ≥ 1. Therefore an MRR of 1 implies that all patients or practices are equally likely to suffer or cause harm, while an MRR of 2 implies an average twofold difference in harm susceptibility.

We assumed that even the most homogeneous population of practices and patients would differ in underlying harm rate to some degree, so chose a minimum MRR of 1.2. We assessed inter-patient variation in harm rate (the tendency for some patients to be more harm prone) MRRs of 1.2 and 2, representing relatively homogeneous and more diverse populations respectively. For practices we present results for MRR = 1.2 only, based on our preliminary analyses which suggested varying MRR among practices had little effect on the precision and power of harm rate estimates (compared with the effect of varying MRR among patients).

### Simulation of CRR scenarios

We generated 1000 random data sets for each of the 864 possible combinations of the different parameter values. Each simulated data set recorded the number of harm incidents “observed” in each patient’s records, within each practice, at each timepoint. Each simulated data sets is intended to represent what may have been produced by an actual review in ‘real life’. We estimated the harm rate at both time points and tested for over a 12-month period for each data set, before averaging over all 1000 sets of results to give the expected estimation error and power for every record review scenario. Average estimation error was calculated using the median, while power was estimated as the percentage of the 1000 data sets where the test of the null hypothesis of no change in the harm rate was significant (P < 0.05). Percentage estimation error was calculated as 100 × 0.5 × median confidence interval width divided by median harm rate estimate. Sampling error in the power and precision estimates was small because of the large number of data sets generated and we therefore for simplicity of presentation we do not show confidence intervals (CIs) around the estimates. We also simulated and analysed data under the null hypothesis of ‘no change’, which allowed us to validate the methodology by checking the type I error rate (results not shown).

### Simulation and modeling assumptions

Because patients could potentially experience more than one harm incident during the review period, the number of detected events was treated as count rather than binary data and simulated and modelled as a Poisson random variable within the framework of a generalised linear model (GLM). In addition, the expectation of variation in underlying harm rates between patients and practices motivated the inclusion of random effects. We therefore simulated and modelled the number of harm incidents as a Poisson generalised linear mixed model (GLMM) with harm rates allowed to vary randomly between practices and patients, assuming a lognormal distribution for the random effects (the Poisson-lognormal model) [26]. The change in harm rate over the 12-month study period was simulated and modeled as a fixed effect and is presented as the percentage reduction in the real harm rate.

Specifically, the number of harm incidents *y*_*ijk*_ detected during review of the records of patient *i* in practice *j* at time *k* was drawn from a Poisson distribution with harm rate *λ*_*ijk*_; that is, *y*_*ijk*_ ~ *Pois*(*λ*_*ijk*_). The effects of time, inter-patient and inter-practice variation influenced the harm rate additively on the log scale such that log(*λ*_*ijk*_) = *α* + *βx*_*ijk*_ + *u*_1*i*_ + *u*_2*j*_ where *α* is the overall mean log harm rate at the first review and *β* is the fixed effect of time. *x* indicates whether record review is taking place at the beginning (*x* = 0) or end (*x* = 1) of the twelve months period, so that the mean log harm rate is *α* at the first and *α* + *β* at the second review. *u*_1_ and *u*_2_ are patient- and practice-level random effects, respectively, that allow harm rate to vary between patients within practices and between practices.*u*_1_ and *u*_2_ are normally distributed with variances *ϕ*|_1_ ^2^ and *ϕ*_2_ ^2^, respectively.

Estimation and significance testing were carried out by fitting a GLM with a Poisson response [27]. This model does not allow adjustment for inter-practice and inter-patient variation (which we know to be present) and we therefore expected the model estimates to be biased. We initially attempted to adjust for both these sources of variation by fitting the same Poisson-lognormal GLMM that was used to simulate the data. However, in preliminary analyses we found that the number of harm incidents was too low, even at the highest harm rate, to allow the GLMM-fitting algorithm to converge on either the inter-practice or the inter-patient variance estimates.

### Statistical analysis

All analyses were performed in R version 2.14.0. GLMs were fitted using the *glm* function, while GLMMs were fitted using the *glmer* function in the *lme4* package [28]. We confirmed that the failure of GLMMs to fit was not specific to the algorithm used by fitting a selection of models by penalised quasi-likelihood (using glmmPQL in R) [29] and Monte Carlo Markov Chain (using MCMCglmm in R) [30]. We assessed the validity of the fitted models by monitoring estimation bias, confidence interval coverage and type I error rate.

A problem encountered when some of datasets were simulated with very low or no harm incidents at one or both visits then the model-fitting algorithm failed to converge and did not yield valid harm rate estimates or p-values. When estimating power, these refractory data sets were counted as yielding non-significant results. For the purpose of estimating CI width, estimates from these failed model fits were excluded from the calculation of the median, except when ≥10% of fits failed, in which case the average CI was not calculated for that CRR scenario. Such data sets were generated only when very low precision (estimation error > ±100%) and power (<20%) were expected, and therefore represent CRR scenarios that should be avoided when aiming to study, measure or monitor the incidence of harm in general practice.

## Results

### Precision of estimated harm rates

**Figure 1:**
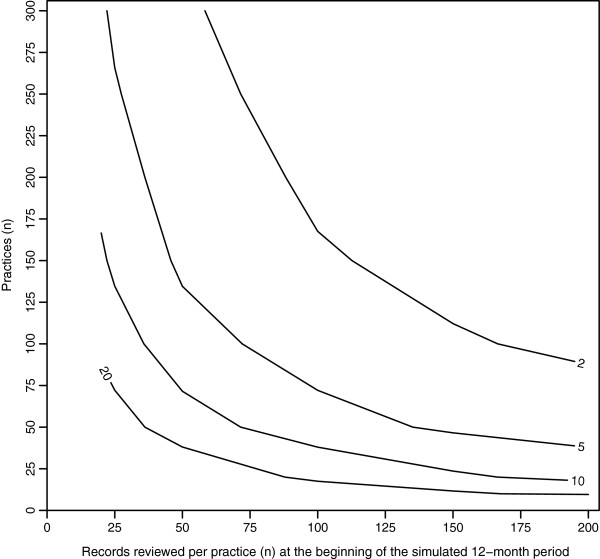
**Clinical record review (CRR) scenarios which yielded harm rate estimates with acceptable precision, e.g. estimation errors < ±25% of the real harm rate (rHR).** The lines and the zone above and to the right of each line represent those CRR scenarios with acceptable precision. Scenarios vary according to number of practices reviewing records, number of records reviewed per practice and the rHR (indicated by numbers on the lines and measured in incidents/100 patients/year. The median rate ratios (MRR) between patients and practices are 2 and 1.2 respectively. The results are for the beginning of the simulated twelve month period.

Table 2 summarizes the estimation errors of selected CRR scenarios’ harm rate estimates. Figure 1 provides a graphical display of all CRR scenarios that yielded harm rate estimates with acceptable precision. Three parameters were varied in these CRR scenarios: (i) the number of practices reviewing records, (ii) number of records reviewed in each practice and (iii) the real harm rate (rHR) prevalent in the patient population at the beginning of the 12-month period specified for this study. All reported results are for CRR scenarios with high inter-patient variation (MRR = 2). CRR scenarios with low inter-patient variation (MRR = 1.2) are presented as supplementary tables and figures (Additional file 1).

**Table 2:** **The precision**^**$ **^**of selected clinical record review (CRR) scenarios’ estimated harm rates**

**Practices (n)**	**Records reviewed per practice (n)**	**Estimation error (%)**^**$**^			
		rHR^*^ **= 2**	rHR^*^ **= 5**	rHR^*^ **= 10**	rHR^*^ **= 20**
1	20	-	-	-	-
	25	-	-	-	-
	50	-	-	-	-
	100	-	-	-	107
	150	-	-	132	88
	200	-	-	107	74
10	20	-	-	107	74
	25	-	146	96	67
	50	-	96	65	46
	100	107	65	46	32
	150	88	54	37	26
	200	74	46	32	23**
20	20	-	107	74	51
	25	-	96	65	46
	50	107	65	46	32
	100	74	46	32	23
	150	60	37	26	18
	200	51	32	**23**	16
50	20	107	67	46	32
	25	96	58	41	29
	50	65	41	29	20
	100	46	29	20	14
	150	37	**23**	17	12
	200	32	20	14	10
100	20	74	46	32	23
	25	65	41	29	20
	50	46	29	20	14
	100	32	20	14	10
	150	26	17	12	8
	200	23	14	10	7
150	20	60	37	26	18
	25	54	33	23	17
	50	37	23	16	12
	100	26	17	12	8
	150	21	13	10	7
	200	18	12	8	6
200	20	52	32	23	16
	25	46	29	20	14
	50	32	20	14	10
	100	23	14	10	7
	150	18	12	8	6
	200	16	10	7	5
250	20	46	29	20	14
	25	41	26	18	13
	50	29	18	13	9
	100	20	13	9	6
	150	17	10	7	5
	200	14	9	6	5
300	20	42	26	18	13
	25	37	23	17	12
	50	26	17	12	8
	100	18	12	8	6
	150	15	10	7	5
	200	13	8	6	4

^$^Precision is expressed as percentage estimation error, averaged across 1000 simulated studies for each CRR scenario.

*rHR (real harm rate): The *actual*, underlying ‘baseline’ harm rate, expressed as number of incidents/100 patients/year. The harm rates estimated by different CRR scenarios are *not* shown.

**Example: In this CRR scenario, 10 practices each reviewed 200 records and the estimation error was ±23%, e.g. within ±23% of the rHR of 20 incidents/100patients/year. The estimation error (%) indicates the proximity between the harm rate estimated by that unique CRR scenario and the rHR. Smaller estimation errors therefore indicate greater precision. We defined acceptable precision as estimation errors < ±25%.

Scenarios vary by numbers of practices reviewing records, number of records reviewed in each practice and real harm rates (rHR)*. The median rate ratios (MRR) between patients and practices are 2 and 1.2 respectively. The results are from the beginning of the simulated 12-month period.

Different combinations of certain parameter values produced CRR scenarios which yielded harm rate estimates with acceptable precision. For a rHR of 20 incidents/100 patients/year, 2000 unique patient records had to be reviewed in total to ensure the harm rate estimate had adequate precision. Table 2 and Figure 1 show that adequate precision was achieved by any combination of numbers of practices and records that yielded approximately 2000 records: ten practices × 200 records each; 20 practices × 100 records each; etc.

For a rHR of 10 rather than 20 incidents/100 patients/year, the total number of records to review increased from 2000 (for example 10 practices × 200 records each, precision ±23%), to 4000 (e.g. 20 practices × 200 records each, precision ±23%) to ensure adequate precision of harm rate estimates. Again, the particular combination of numbers of practices and records required to reach this critical number of records was not important. Similarly, for rHR of 5 and 2 incidents/100 patients/year, a total of 7500 (e.g. 50 practices × 150 records each, precision ±23%) and 20000 records (e.g. 100 practices × 200 records, precision ±23%) had to be reviewed to ensure adequate precision of harm rate estimates (Table 2, Figure 1). Put simply, lower real harm rates required CRR scenarios with greater numbers of records to be reviewed to ensure their harm rate estimates still had adequate precision. We noted that the constant factor across all scenarios giving adequate precision was the expected number of harm incidents, which was 100.

### CRR scenarios’ power to detect reductions in harm

**Figure 2:**
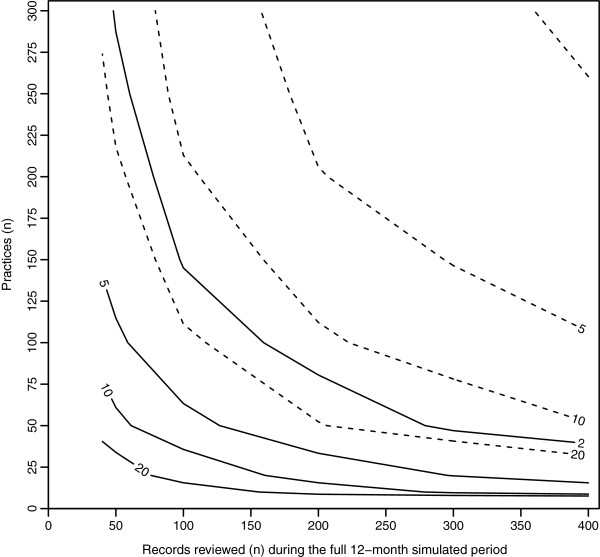
**Clinical record review (CRR) scenarios with adequate power (≥80%) to detect a 50% (solid line) or 20% (dashed line) reduction in real harm rates (rHR) over a 12-month period.** The lines and the zone above and to the right of each line represent CRR scenarios with adequate power. Scenarios vary according to number of practices reviewing records, number of records reviewed per practice and the rHR (indicated by numbers on the lines and measured in incidents/100 patients/year. The median rate ratios (MRR) between patients and between practices were 2 and 1.2 respectively.

Table 3 shows the power (adequate or inadequate) of selected CRR scenarios, while Figure 2 graphically displays all CRR scenarios with adequate power to detect reductions in harm over the specified period of twelve months. Four parameters were varied in these CRR scenarios: (i) the number of practices reviewing records, (ii) number of records reviewed in each practice; (iii) the real harm rate (rHR) prevalent in the patient population at the beginning of the twelve month period; and (iv) the reduction in the rHR (50% or 20%).

**Table 3:** Power (%)* of selected clinical record review (CRR) scenarios to detect a reduction (R) in the real harm rate (rHR) over a twelve month period

**Practices (n)**	Records reviewed (n)**	**Power (%)**							
		**rHR = 2**		**rHR = 5**		**rHR = 10**		**rHR = 20**	
		**R = 50%**	**R = 20%**	**R = 50%**	**R = 20%**	**R = 50%**	**R = 20%**	**R = 50%**	**R = 20%**
1	40	0	0	0	0	0	0	0	0
	50	0	0	0	0	0	0	0	0
	100	0	0	0	0	0	0	1	1
	200	0	0	0	0	2	1	9	5
	300	0	0	0	0	3	2	19	7
	400	0	0	1	1	11	4	27	7
10	40	0	0	1	0	8	4	30	8
	50	0	0	2	1	16	5	37	8
	100	1	0	14	5	38	7	64	14
	200	9	4	36	8	66	13	93	22
	300	21	5	57	10	84	16	98	32
	400	28	8	65	12	91	22	100	41
20	40	0	0	10	4	28	7	53	11
	50	0	0	17	6	37	8	66	15
	100	10	4	37	10	63	13	93	24
	200	28	7	67	13	91	22	100	38
	300	43	9	81	19	98	31	100	54
	400	56	11	92	20	100	40	100	69
50	40	9	3	34	8	65	13	93	24
	50	14	5	45	10	75	16	96	28
	100	36	10	74	16	96	25	100	48
	200	64	12	97	27	100	48	100	79
	300	**84**^**$**^	**18**^**$**^	100	41	100	64	100	92
	400	94	21	100	48	100	79	100	97
100	40	31	6	64	11	92	20	100	40
	50	35	10	76	14	96	29	100	53
	100	66	12	97	26	100	47	100	77
	200	90	22	100	48	100	76	100	96
	300	98	34	100	64	100	92	100	100
	400	100	38	100	78	100	97	100	100
150	40	45	10	84	16	99	33	100	56
	50	51	11	89	21	100	38	100	64
	100	82	15	100	39	100	64	100	91
	200	99	32	100	64	100	91	100	100
	300	100	46	100	81	100	99	100	100
	400	100	57	100	92	100	100	100	100
200	40	53	12	91	23	100	38	100	68
	50	64	12	96	28	100	47	100	77
	100	92	22	100	49	100	78	100	97
	200	100	36	100	79	100	96	100	100
	300	100	56	100	92	100	100	100	100
	400	100	67	100	96	100	100	100	100
250	40	64	14	98	25	100	49	100	77
	50	76	13	99	36	100	58	100	85
	100	97	30	100	55	100	86	100	99
	200	100	50	100	87	100	99	100	100
	300	100	67	100	95	100	100	100	100
	400	100	79	100	99	100	100	100	100
300	40	74	15	99	31	100	55	100	84
	50	82	18	99	37	100	64	100	90
	100	98	29	100	65	100	91	100	100
	200	100	54	100	91	100	100	100	100
	300	100	73	100	99	100	100	100	100
	400	100	85	100	100	100	100	100	100

*Power below the type I error rate of 5% is possible because analyses where the estimates failed to converge are counted as failure to detect change in the harm rate.

**The total number of records reviewed during the twelve month period is shown. Each patient record was reviewed twice during this time.

^$^Example: In this CRR scenario, 50 practices each reviewed 300 records (150 at the beginning and 150 at the end of twelve months) and had a baseline rHR of 2 incidents/100patients/year. Reductions of 50% and 20% over a twelve month period were detected with 84% (adequate) and 18% (inadequate) power respectively.

Scenarios vary by numbers of practices reviewing records, number of records reviewed in each practice and rHR. Median rate ratios (MRR) between patients and practices are 2 and 1.2 respectively.

Similar to precision, different combinations of parameter values produce CRR scenarios with adequate power. For a rHR of 20 incidents/100 patients/year reduced by 50%, any CRR scenario that required 2000 record reviews had adequate power (Table 3): 10 practices each reviewing 100 records twice; 20 practices each reviewing 50 records twice; 50 practices each reviewing 20 records twice. Figure 2 shows many other potential parameter value combinations which also produced CRR scenarios with adequate power.

Smaller reductions and lower baseline (real) harm rates required CRR scenarios with increasing numbers of records to ensure adequate power. For a rHR of 10 instead of 20 incidents/100 patients/year in the aforementioned example, the total number of records to review increased from 2000 to 3000 to ensure a 50% reduction was detected with acceptable power. For baseline harm rates of 5 or 2 incidents/100 patients/year, the total number of records reviewed increased further to approximately 6000 and 15000 respectively (Table 3, Figure 2). As with the results for precision, the critical factor in achieving adequate power to detect a 50% reduction in harm rate was the number of harm incidents that were expected to be observed, which was almost invariant at 75–100.

Detection of a more modest 20% reduction required a fivefold increase in the number of reviewed records. For the same set of rHRs of 20, 10, 5 or 2 incidents/100 patients/year, CRR scenarios required reviewing 12 000, 25 000, 45 000 and 120 000 records, respectively. This translates into the relatively simple rule that there will be adequate power to detect a 20% reduction in harm rate if approximately 600 harm incidents are expected to be observed.

### A formula for precision and power

**Figure 3:**
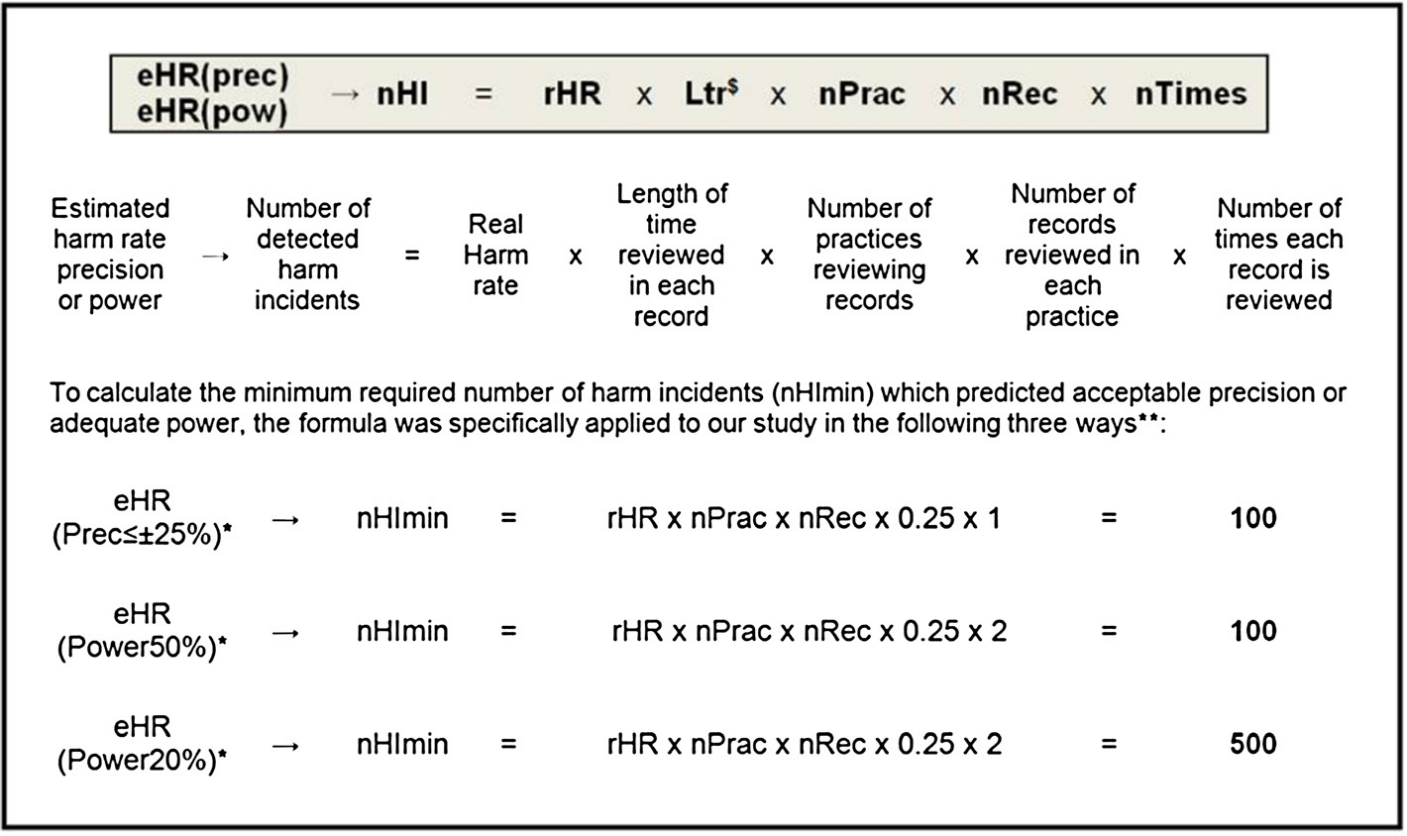
**A formula to express the relationship between the parameters of CRR scenarios and their numbers of detected harm incidents, which is associated with the precision and power of estimated harm rates. **^$^We specified a three month period of review in each record for the purposes of this study. In our examples this is expressed as 0.25 years. Increasing the review period from three to twelve months would have resulted in a fourfold reduction in the number of records each practice had to review. *The levels of precision and power we selected for the purposes of this study. **The values of rHR, nPrac and nRec are taken from the ‘lines’ in Figures 1 and 2.

Levels of precision and power were mainly determined by the number of harm incidents expected in any given CRR scenario. The number of harm incidents is in turn determined by the real harm rate (rHR) multiplied by the total time reviewed, across all patients, during the complete CRR process. The time and effort required to complete the CRR process is a product of: 1. the period of time reviewed in each record; 2. number of records reviewed per practice; 3. number of practices reviewing records; and 4. the number of times each record is reviewed. The relationship between the number of detected harm incidents and CRR parameters can be simplified and expressed as a formula (Figure 3).

Substituting the numbers which constitute the ‘lines’ in Figures 1 and 2 (the number of practices reviewing records, number of records being reviewed per practice and the real harm rates) into this formula resulted in a ‘constant’ number of detected harm incidents. This ‘constant’ is the minimum number of harm incidents which had to be detected during any CRR process to ensure acceptable precision or adequate power of its estimated harm rates.

We found that any CRR scenario (e.g. any combination of record review parameters and parameter values) that result in at least 100 harm incidents being detected will have harm rate estimates with acceptable precision (as defined by us). Reductions of ≥ 50% in harm were detected with adequate power by any CRR scenario which ensured at least 100 harm incidents were detected during the specified time period (twelve months). However, to detect a 20% reduction, CRR scenarios had to allow detection of a minimum of 500 harm incidents.

### Estimating harm in a single general practice

It is evident from Figures 1 and 2 that no combination of parameter values yielded a global harm rate estimate with acceptable precision or adequate power for a single general practice. For a practice that reviewed 200 records and assumed a relatively high rHR of 20 incidents/100 patients/year, the estimated error was ±74% (Table 2). If the practice reviewed the 200 records for a second time, 12 months later, their CRR strategy would have 27% and 7% power to detect a 50% and 20% reduction in the rHR respectively (Table 3). Using the formula (Figure 3), and assuming a rHR of 20 incidents/100patients/year and a 50% reduction in harm, we calculated that a practice would have to review a minimum of 2000 records to ensure their estimated global harm rate had acceptable precision and adequate power.

### Inter-patient variation and bias

Bias was a substantial influence on the harm rate estimates of those CRR scenarios with high inter-patient variation (MRR = 2), being typically around 30% (Additional file 1: Table S3; median bias across all scenarios: 28%; interquartile range: 23% to 30%). For example, the CRR scenario in Table 2 with a rHR of 20 incidents/100 patients/year and 20 practices each reviewing 100 records had adequate precision (estimation error = 24%). However, this estimation error does not include bias, which for this CRR scenario was 30%. Combining precision and bias, a typical harm rate estimate for this CRR scenario would be 26 (95% CI 19.5-32.5) incidents/100 patients/year. Where inter-patient variation was low (MRR = 1.2), bias was insignificant or absent (Additional file 1: Table S3; median bias across all scenarios: 0%; interquartile range: -4% to 2%). Inter-patient variation had no significant impact on power to detect a reduction in harm rates.

## Discussion

In this study we described a number of parameters which may affect the precision and power of CRR harm estimates in general practice. We combined a wide range of different parameter values into different CRR scenarios and used computer simulation to establish which ones would yield harm rate estimates with acceptable precision and adequate power. From this, we derived a formula which we used to calculate the minimum number of harm incidents that had to be detected during any CRR process to ensure the harm estimates had acceptable precision and adequate power. We found that any CRR scenario which detected a minimum of 100 harm incidents would have harm rate estimates with the level of precision we pre-specified. Using the formula and our simulated data, we calculated that detecting a 50% and 20% reduction in harm with acceptable power would require CRR scenarios to detect at least 100 and 500 harm incidents respectively, over a given period of time.

The practical implication of the CRR scenarios which assures harm rate estimates with acceptable precision (as defined by us) is that approximately 2000 records (assuming a high baseline harm rate) increasing to 20 000 records (assuming a low harm rate) would have to be reviewed. If the aim of the CRR is to detect changes in harm rates with adequate power over time as many as 120 000 records may have to be reviewed, depending on the prevalence of the harm in the patient population of interest. Different parameter values can be combined into different CRR scenarios by health care researchers, clinicians, policy makers and others to fit their aims and resources. By applying our formula, they could ensure the harm estimates of these potential different CRR scenarios will have adequate precision and acceptable power.

### Comparison with the literature

The vast majority of studies with a CRR methodology aim to detect either patient safety incidents (PSIs) in general, or more specific subsets of PSIs such as harm, adverse drug reactions or errors, to estimate a harm rate for a defined geographical location or clinical department at specified points in time. Our non-systematic search of the relevant international literature [1,3] did not uncover a single study in which the precision of these reported rates was either considered or documented. In addition, none seem to have explicitly considered the required parameter values of their CRR method. Instead, the size of the patient record samples seemed determined only by resources, time and feasibility concerns. While this observation does not necessarily imply that all previous harm rate estimates were imprecise, our findings suggest that any CRR which detected less than 100 harm incidents may not have had adequate precision (as defined by us).

To illustrate this point further, we provide three practical examples. Example one: Singh and colleagues measured the adverse drug event rate amongst older patients with established cardiovascular disease by reviewing a 12-month period in 393 pre-screened trigger positive records from six general practices in the UK [18]. They found 232 adverse drug events, of which 92 were judged preventable, with an estimated rate of 24.6 preventable adverse drug incidents/100patients/year. Applying our formula to their CRR method and findings suggest their estimated rate has adequate precision (as defined by us). Example two: Gaal and colleagues reviewed 1000 unique medical records in Dutch general practice, over a 12 month period, and estimated a rate of 21.1 patient safety incidents/100patients/year (CI 18.5-24.1), and 5.8 harm events/100patients/year [19]. Applying our formula to their CRR method and findings suggest the estimated PSI rate is precise but the harm rate estimate may not be. Example three: De Wet and Bowie reviewed a 12-month period in each of 100 records randomly sampled in five participating practices in Scotland [17]. Overall, 64 PSIs were found, which is less than the 100 harm incidents our formula suggests for acceptable precision.

Only a tiny minority of studies using the CRR method has aimed to measure reductions in harm rates over time. They were all conducted in secondary care settings and to our knowledge there has been none in primary care. Carter described the experiences of a hospital in the UK with the global trigger tool over a five year period [31]. While it ‘appeared’ that the incidence of ‘more serious’ events reduced and ‘more minor’ harm incidents increased, the changes were not quantified. Landrigan and colleagues’ review of 2341 admissions to 10 USA hospitals over a six year period was the largest study of its type when it was published, but failed to detect a significant reduction in the rate of harm during this period [8,32]. Applying our formula (which suggests detecting a 20% change in harm would require a CRR to detect at least 500 preventable harm incidents) to their findings suggests at least two possibilities: either there was no reduction in harm, or there was a small reduction but the sample was insufficiently powered to detect it.

Our simulations represent ‘best case’ scenarios and likely underestimate the amount of records that may have to be reviewed. While we know that a substantial proportion of PSIs may not be preventable because they originated in different settings, are recognized as side effects of appropriate treatment or are dependent on patient factors, this was not directly controlled for in our simulations. Current estimates suggest between 10 and 50% of detected harm incidents may be preventable [9,18,33,34]. Therefore, when researchers or reviewers attempt to measure reductions in harm over time, they have to remove, or at the very least consider, what proportion of the detected harm incidents are likely to be’non-preventable’. Otherwise, the observed reduction will appear ‘smaller’ (as a percentage) than it actually was, and their CRR scenario’s power to detect the change will also be decreased.

To illustrate this point further, consider the study conducted by Takata and colleagues as a practical example. They detected 107 adverse drug events, of which 24 (22%) were judged preventable in their review of 960 paediatric records from 12 USA hospitals [16]. If they aimed to reduce the number of preventable incidents by an ambitious 50% (e.g. a reduction from 24 to 12 incidents) over a given period of time, this reduction would ‘only’ be 11.2% of their overall ADE rate. Our findings suggest this would require a CRR of many thousands of records, and certainly much more than if their aim had been a reduction of 50% in the overall rate.

### Potential application of findings

There is considerable political and policy interest in a measure to reliably quantify and then track rates of harm in primary care records over time. The ideal attributes of such a measure are that it should be: relevant; valid; reliable; discriminative; credible; timely; feasible; accessible; and actionable [35]. CRR has most of these attributes, but may be limited by feasibility concerns. Our findings are the first known attempt to quantify the minimum CRR parameter values which impact on feasibility (e.g. number of practices reviewing records and number of records reviewed per practice) and may therefore help to inform the discussion and planning of health care policy makers and leaders who are interested in measuring harm in general practice.

While our findings suggest a single general practice cannot feasibly measure its rate of harm with acceptable precision or adequate power, we provided many CRR scenarios that would yield harm rate estimates with adequate precision and acceptable power if implemented at national or regional level and a formula to test any other proposed CRR adaptations.

At national level, there are 1003 general medical practices in Scotland [22]. Our findings suggest that if at least 300 practices each reviewed 25 records twice over a given period of time (say 12 months), the CRR sample yield harm rate estimates with acceptable precision and would have adequate power to detect a 50% reduction in ‘any’ assumed baseline harm rate if it occurred during this period. Smaller changes in harm rates could be detected if every practice in Scotland participated, although engagement would likely have be sought through contractual incentivisation.

Let us consider two examples at the regional level. Example one: A Scottish regional Health Board with 100 general medical practices aims to estimate their harm rate with acceptable precision. If they assume a real (baseline) harm rate of 10 incidents/100 patients/year, our formula indicates that each practice will have to review 50 records to achieve this aim. If the Health Board assume a lower harm rate of 5 incidents/100patients/year or selects a less harm prone patient population, each practice will have to review 100 records to achieve a harm rate estimate with acceptable precision. Example two: A Scottish regional Health Board wants to estimate the harm rate in their region which has 57 general practices. If they assume a baseline harm rate of 5 incidents/100 patients/year, each practice would have to review 150 records to estimate the harm rate with acceptable precision.

Measuring at regional and national level will require substantial investment in training and support, allocation of additional resources and protected time for clinician reviewers.

### Strengths and limitations

Our findings were derived by aggregating the results of multiple simulated data sets for different CRR scenarios derived from predefined parameters and parameter values. Our assumptions about these parameter values were informed by practical experience and available literature. Given that the available evidence of harm prevalence and preventability varies widely, our choices of harm rates and potential reductions in harm are therefore likely to include overestimations of incidence and reductions.

Our statistical method allowed simulation of complex scenarios, but the data remains simulated and at best a simplified and imprecise presentation of reality. We accepted the principle that the same patient may suffer more than one incident during a review period. This meant that data had to be treated as ‘count’ rather than binary. The consequences were that harm rates had to be expressed as rates (i.e. incidents/100 patients/year) and not percentages, and sensitivity, specificity and predictive value could not be calculated. Potential inter-rater bias and intra-rater error (inconsistency) were accounted for by ‘including’ it as part of the inter-practice variation in harm rate. We assumed the same patients’ records were reviewed at the beginning and end of the study period. This reduced inter-patient variation and increased power.

We also identified a problem of substantial positive bias in harm rate estimates where there are high levels of inter-patient variation. The standard approach of quantifying and adjusting for inter-patient variation was not feasible due to the very low numbers of harm incidents in some CRR scenarios. These results suggest that estimates of harm rates from CRRs could contain unquantifiable upward bias due to unknown levels of inter-patient variation. This is a problem that will affect real studies and not an artefact of our analysis. It is a consequence of making estimates from multilevel data where the numbers of events are too small to allow the multilevel effects to be adjusted for. The sample sizes required to adjust for these effects were beyond the realistic range explored here and may be unfeasible. The implications of this inability to estimate random effects go beyond bias in harm rate estimates to scenarios where variation between practices is of primary interest rather than simply a parameter to be adjusted for. If the aim of CRR was to determine whether some practices have significantly higher harm rates than others, or if the harm rates of some practices are changing (increasing or decreasing) faster than others, considerably larger numbers of patient safety incidents would have to detected than in our simulations. This would require increasing the number of records reviewed, lengthening the review period and/or selecting an unusually harm-prone population of patients.

### Future research

We simulated CRR to detect changes over a single time period. In our scenarios power was maximised by reviewing records at only two time points - the beginning and end of a 12-month period. However, many patient safety programs may not be time-limited or will measure harm at multiple time points. The availability of data at additional time points will allow the detection of trends. Monte Carlo simulations could be used in future research to optimise experimental design for such longitudinal scenarios.

The relationship between measurement and improvement, and the challenge of ‘getting one to follow the other’ has previously been described [35]. We still do not know which interventions can successfully improve patient safety in general practice. What little evidence there is suggests successful interventions will likely require a multi-method approach, rigorous evaluation and small, local clinician-led pilots [36]. Future research should therefore examine the utility of CRR as a learning and improvement tool, ‘…working on the nuts and bolts of how we turn measurement for improvement into tangible change in practice… [35]’. Other potential research questions include: the effects of inter-patient and practice variation on estimated harm rates; and what the ideal mixture of parameter values (number practices, records reviewed in each practice and review time per record) are to detect the minimum number of harm incidents to ensure acceptable precision and adequate power. Finally, our statistical model and formula needs to be validated further through practical application.

## Conclusion

This study is the first known attempt to describe the minimum parameter values of any CRR which will ensure its harm rate estimates have adequate precision and adequate power. We derived a formula which allows calculation of the minimum number of harm incidents which have to be detected with a CRR to ensure adequate precision and acceptable power. Our findings have practical implications for health care decision-makers, leaders and researchers aiming to measure harm at regional or national level.

## Ethical review

The study did not require ethical approval.

## Competing interests

The authors declare that they have no competing interests

## Study funding

The NHS Education for Scotland Patient Safety Multi-Professional Group.

## Pre-publication history

The pre-publication history for this paper can be accessed here:

http://www.biomedcentral.com/1471-2288/13/39/prepub

## Supplementary Material

Additional file 1: Table S1 The precision$ of selected clinical record review (CRR) scenarios’ estimated harm rates. Scenarios vary by numbers of practices reviewing records, number of records reviewed in each practice and real harm rates (rHR)*. The median rate ratios (MRR) between patients** and practices are 1.2 and 1.2 respectively. The results are from the beginning of the simulated 12-month period. **Figure S1**. Clinical record review (CRR) scenarios which yielded harm rate estimates with acceptable precision, e.g. estimation errors < ±25% of the real harm rate (rHR). The lines and the zone above and to the right of each line represent those CRR scenarios with acceptable precision. Scenarios vary according to number of practices reviewing records, number of records reviewed per practice and the rHR (indicated by numbers on the lines and measured in incidents/100 patients/year. The median rate ratios (MRR) between patients and practices are 1.2 and 1.2 respectively. The results are for the beginning of the simulated twelve month period. **Table S2**. Power (%)* of selected clinical record review (CRR) scenarios to detect a reduction (R) in the real harm rate (rHR) over a twelve month period. Scenarios vary by numbers of practices reviewing records, number of records reviewed in each practice and rHR. Median rate ratios (MRR) between patients and practices are 1.2 and 1.2 respectively. **Figure S2**. Clinical record review (CRR) scenarios with adequate power (≥80%) to detect a 50% (solid line) or 20% (dashed line) reduction in real harm rates (rHR) over a twelve month period. The lines and the zone above and to the right of each line represent CRR scenarios with adequate power. Scenarios vary according to number of practices reviewing records, number of records reviewed per practice and the rHR (indicated by numbers on the lines and measured in incidents/100 patients/year. The median rate ratios (MRR) between patients and between practices were 1.2 and 1.2 respectively. **Table S3**. The 95% confidence interval coverage and bias of selected clinical record review (CRR) scenarios’ estimated harm rates. Coverage estimates that are significantly different from 95% are underlined. Scenarios vary by numbers of practices reviewing records, number of records reviewed in each practice and real harm rates (rHR)*. The median rate ratios (MRR) between patients and practices are 1.2 and 1.2 respectively. The results are from the beginning of the simulated 12-month period. **Table S4**. The 95% confidence interval coverage and bias of selected clinical record review (CRR) scenarios’ estimated harm rates. Coverage estimates that are significantly different from 95% are underlined. Scenarios vary by numbers of practices reviewing records, number of records reviewed in each practice and real harm rates (rHR)*. The median rate ratios (MRR) between patients and practices are 2 and 1.2 respectively. The results are from the beginning of the simulated 12-month period. Click here for file
