# Supplementary material for: Can we quantify harm in general practice records? An assessment of precision and power using computer simulation
Source: BMC Med Res Methodol. 2013 Mar 13;13:39. doi: 10.1186/1471-2288-13-39 (PMC3635932; doi:10.1186/1471-2288-13-39)
Supplement: Additional file 1: Table S1 — The precision$ of selected clinical record review (CRR) scenarios’ estimated harm rates. Scenarios vary by numbers of practices reviewing records, number of records reviewed in each practice and real harm rates (rHR)*. The median rate ratios (MRR) between patients** and practices are 1.2 and 1.2 respectively. The results are from the beginning of the simulated 12-month period. Figure S1. Clinical record review (CRR) scenarios which yielded harm rate estimates with acceptable precision, e.g. estimation errors < ±25% of the real harm rate (rHR). The lines and the zone above and to the right of each line represent those CRR scenarios with acceptable precision. Scenarios vary according to number of practices reviewing records, number of records reviewed per practice and the rHR (indicated by numbers on the lines and measured in incidents/100 patients/year. The median rate ratios (MRR) between patients and practices are 1.2 and 1.2 respectively. The results are for the beginning of the simulated twelve month period. Table S2. Power (%)* of selected clinical record review (CRR) scenarios to detect a reduction (R) in the real harm rate (rHR) over a twelve month period. Scenarios vary by numbers of practices reviewing records, number of records reviewed in each practice and rHR. Median rate ratios (MRR) between patients and practices are 1.2 and 1.2 respectively. Figure S2. Clinical record review (CRR) scenarios with adequate power (≥80%) to detect a 50% (solid line) or 20% (dashed line) reduction in real harm rates (rHR) over a twelve month period. The lines and the zone above and to the right of each line represent CRR scenarios with adequate power. Scenarios vary according to number of practices reviewing records, number of records reviewed per practice and the rHR (indicated by numbers on the lines and measured in incidents/100 patients/year. The median rate ratios (MRR) between patients and between practices were 1.2 and 1.2 respectively. Table S3. The 95% confidence i [file 1471-2288-13-39-S1.doc]

**Can we quantify harm in general practice records? An assessment of precision and power using computer simulation**

**Additional file 1: Supplementary tables and figures**

**Supplementary Table 1. The precision^$^ of selected clinical record review (CRR) scenarios’ estimated harm rates. Scenarios vary by numbers of practices reviewing records, number of records reviewed in each practice and real harm rates (rHR)*. The median rate ratios (MRR) between patients** and practices are 1.2 and 1.2 respectively. The results are from the beginning of the simulated 12-month period.**

| Practices (n) | Records reviewed per practice (n) | Estimation error (%) | | | |
| --- | --- | --- | --- | --- | --- |
|  |  | rHR=2 | rHR=5 | rHR=10 | rHR=20 |
| 1 | 20 25 50 100 150 200 | - - - - - - | - - - - - - | - - - - 122 99 | - - - 99 80 66 |
| 10 | 20 25 50 100 150 200 | - - - 99 76 66 | - 133 85 59 47 41 | 99 85 59 41 33 28 | 66 59 41 29 23 20 |
| 20 | 20 25 50 100 150 200 | - - 99 66 53 45 | 99 85 59 41 33 28 | 66 59 41 29 23 20 | 45 41 28 20 16 14 |
| 50 | 20 25 50 100 150 200 | 99 85 59 41 33 29 | 59 52 37 26 21 18 | 41 36 26 18 15 13 | 29 26 18 13 10 9 |
| 100 | 20 25 50 100 150 200 | 66 59 41 29 23 20 | 41 37 25 18 15 13 | 29 26 18 13 10 9 | 20 18 13 9 7 6 |
| 150 | 20 25 50 100 150 200 | 53 47 33 23 19 16 | 33 30 21 15 12 10 | 23 21 15 10 8 7 | 16 15 10 7 6 5 |
| 200 | 20 25 50 100 150 200 | 45 41 28 20 16 14 | 29 26 18 13 10 9 | 20 18 13 9 7 6 | 14 13 9 6 5 4 |
| 250 | 20 25 50 100 150 200 | 41 36 26 18 15 13 | 25 23 16 11 9 8 | 18 16 11 8 7 6 | 13 11 8 6 5 4 |
| 300 | 20 25 50 100 150 200 | 37 33 23 16 13 12 | 23 21 15 10 8 7 | 16 15 10 7 6 5 | 12 10 7 5 4 4 |

^$^Precision is expressed as percentage estimation error, averaged across 1000 simulated studies for each CRR scenario.

*rHR (real harm rate): The *actual*, underlying ‘baseline’ harm rate, expressed as number of incidents/100 patients/year. The harm rates estimated by different CRR scenarios are *not* shown.

**Inter-patient variation affected precision and power calculations differently. Increasing inter-patient variation in our model from low (MMR=1.2) to high (MMR=2) had seemingly contradictory results, depending on whether the aim was to estimate precision or power (supplementary Tables 1 and 2). High inter-patient variation (MRR=2) increased the total number of records that had to be reviewed to achieve adequate precision by approximately 20% but decreased the total number by approximately 30% to achieve acceptable power relative to low inter-patient variation (MRR of 1.2). This gain in power appears to be real rather than artefactual, based on our observation of accurate type 1 error, insignificant bias, and accurate confidence interval coverage in estimation and testing of change in harm rate over time. The implications of neglecting to adjust for inter-patient variation therefore appear to be lower precision and upward bias in harm rate estimates, and higher power in detecting changes over time in harm rates.

**Supplementary figure 1.** Clinical record review (CRR) scenarios which yielded harm rate estimates with acceptable precision, e.g. estimation errors <±25% of the real harm rate (rHR). The lines and the zone above and to the right of each line represent those CRR scenarios with acceptable precision. Scenarios vary according to number of practices reviewing records, number of records reviewed per practice and the rHR (indicated by numbers on the lines and measured in incidents/100 patients/year. The median rate ratios (MRR) between patients and practices are 1.2 and 1.2 respectively. The results are for the beginning of the simulated twelve month period.

**Supplementary Table 2.** Power (%)* of selected clinical record review (CRR) scenarios to detect a reduction (R) in the real harm rate (rHR) over a twelve month period. Scenarios vary by numbers of practices reviewing records, number of records reviewed in each practice and rHR. Median rate ratios (MRR) between patients and practices are 1.2 and 1.2 respectively.

| Practices (n) | Records reviewed (n)** | Power (%) | | | | | | | | | | | |
| --- | --- | --- | --- | --- | --- | --- | --- | --- | --- | --- | --- | --- | --- |
|  |  | rHR=2 | | | rHR=5 | | | rHR=10 | | | rHR=20 | | |
|  |  | R=50% | R=20% | R=0% | R=50% | R=20% | R=0% | R=50% | R=20% | R=0% | R=50% | R=20% | R=0% |
| 1 | 40 50 100 200 300 400 | 0 0 0 0 0 0 | 0 0 0 0 0 0 | 0 0 0 0 0 0 | 0 0 0 0 0 0 | 0 0 0 0 0 0 | 0 0 0 0 0 0 | 0 0 0 0 2 4 | 0 0 0 0 0 3 | 0 0 0 0 1 1 | 0 0 1 4 13 22 | 0 0 0 3 4 6 | 0 0 0 3 3 5 |
| 10 | 40 50 100 200 300 400 | 0 0 0 4 13 19 | 0 0 0 2 4 6 | 0 0 0 2 4 4 | 0 1 8 32 43 53 | 0 0 4 7 10 9 | 0 1 2 4 4 6 | 4 9 29 56 71 83 | 2 3 8 12 15 19 | 2 4 5 5 4 6 | 22 27 54 84 94 98 | 6 7 12 17 26 32 | 4 4 5 5 4 6 |
| 20 | 40 50 100 200 300 400 | 0 0 5 21 36 46 | 0 0 2 6 8 10 | 0 0 2 5 5 6 | 4 8 27 53 73 84 | 2 3 7 12 15 20 | 2 2 4 5 4 5 | 22 28 52 86 96 99 | 8 9 10 19 26 31 | 4 4 5 5 4 3 | 44 54 86 98 100 100 | 10 11 18 33 44 59 | 5 5 5 4 5 4 |
| 50 | 40 50 100 200 300 400 | 4 8 29 54 72 84 | 2 4 9 12 14 16 | 1 3 4 4 4 5 | 27 35 64 90 99 100 | 6 8 10 23 32 42 | 3 4 4 6 5 5 | 54 65 90 100 100 100 | 12 13 21 39 57 70 | 5 4 5 5 6 4 | 83 90 100 100 100 100 | 17 23 40 68 83 91 | 5 5 6 5 4 6 |
| 100 | 40 50 100 200 300 400 | 22 28 56 84 96 99 | 8 8 12 17 24 31 | 4 4 6 5 4 5 | 58 64 91 99 100 100 | 11 12 24 41 54 66 | 5 5 6 4 5 4 | 84 91 100 100 100 100 | 19 23 40 64 84 93 | 4 5 6 5 4 6 | 99 100 100 100 100 100 | 31 38 67 92 99 100 | 6 4 5 5 5 6 |
| 150 | 40 50 100 200 300 400 | 33 42 70 95 100 100 | 9 8 13 26 38 44 | 4 4 4 4 6 4 | 72 82 99 100 100 100 | 14 17 31 53 73 81 | 5 4 5 5 4 6 | 95 98 100 100 100 100 | 26 30 56 83 95 99 | 6 6 6 4 4 4 | 100 100 100 100 100 100 | 45 55 82 98 100 100 | 5 5 6 4 5 6 |
| 200 | 40 50 100 200 300 400 | 48 53 85 99 100 100 | 10 12 18 32 48 56 | 5 6 5 5 5 6 | 85 92 100 100 100 100 | 18 22 39 68 80 93 | 5 4 4 5 6 6 | 98 100 100 100 100 100 | 33 40 66 92 99 100 | 5 6 4 5 5 5 | 100 100 100 100 100 100 | 57 68 91 100 100 100 | 4 4 5 6 6 4 |
| 250 | 40 50 100 200 300 400 | 53 60 92 100 100 100 | 12 13 24 38 55 68 | 4 5 4 4 4 5 | 92 97 100 100 100 100 | 21 25 47 78 90 97 | 6 6 4 6 6 3 | 100 100 100 100 100 100 | 42 46 78 97 100 100 | 6 5 5 6 5 4 | 100 100 100 100 100 100 | 70 77 96 100 100 100 | 5 6 5 5 4 6 |
| 300 | 40 50 100 200 300 400 | 60 72 94 100 100 100 | 10 15 26 44 63 75 | 5 4 5 5 6 6 | 96 98 100 100 100 100 | 25 33 53 82 95 99 | 5 4 5 4 6 6 | 100 100 100 100 100 100 | 46 56 83 98 100 100 | 5 5 5 4 6 5 | 100 100 100 100 100 100 | 75 86 98 100 100 100 | 7 4 6 5 5 5 |

*Power below the type I error rate of 5% is possible because analyses where the estimates failed to converge are counted as failure to detect change in the harm rate.

**The total number of records reviewed during the twelve month period is shown. Each patient record was reviewed twice during this time.

**Supplementary figure 2.** Clinical record review (CRR) scenarios with adequate power (≥80%) to detect a 50% (solid line) or 20% (dashed line) reduction in real harm rates (rHR) over a twelve month period. The lines and the zone above and to the right of each line represent CRR scenarios with adequate power. Scenarios vary according to number of practices reviewing records, number of records reviewed per practice and the rHR (indicated by numbers on the lines and measured in incidents/100 patients/year. The median rate ratios (MRR) between patients and between practices were 1.2 and 1.2 respectively.

**Supplementary Table 3**. The 95% confidence interval coverage and bias of selected clinical record review (CRR) scenarios’ estimated harm rates. Coverage estimates that are significantly different from 95% are underlined. Scenarios vary by numbers of practices reviewing records, number of records reviewed in each practice and real harm rates (rHR)*. The median rate ratios (MRR) between patients and practices are 1.2 and 1.2 respectively. The results are from the beginning of the simulated 12-month period.

| Practices (n) | Records reviewed per practice (n) | 95% CI coverage (%), bias (%) | | | |
| --- | --- | --- | --- | --- | --- |
|  |  | rHR=2 | rHR=5 | rHR=10 | rHR=20 |
| 1 | 20 25 50 100 150 200 | - - - - - - | - - - - - - | - - - - 94, 7 95, 0 | - - - 95, 0 92, 7 91, 0 |
| 10 | 20 25 50 100 150 200 | - - - 96, 0 94, -7 95, 0 | - 96, -4 97, -4 94, 4 93, 1 92, 4 | 96, 0 95, -4 95, 4 91, 4 92, 4 90, 2 | 95, 0 95, 4 92, 4 90, 4 91, 4 87, 3 |
| 20 | 20 25 50 100 150 200 | - - 96, 0 95, 0 94, 0 94, 0 | 96, 0 97, -4 96, 4 92, 4 92, 4 92, 2 | 94, 0 95, 4 92, 4 92, 4 92, 3 90, 4 | 92, 0 92, 4 93, 2 92, 3 87, 3 87, 3 |
| 50 | 20 25 50 100 150 200 | 95, 0 97, -4 95, 4 93, 4 93, 1 92, 4 | 95, 4 94, 2 93, 6 93, 4 91, 5 90, 4 | 93, 4 95, 2 95, 4 91, 3 90, 4 91, 3 | 93, 4 94, 4 92, 3 90, 4 84, 3 81, 3 |
| 100 | 20 25 50 100 150 200 | 96, 0 95, 4 94, 4 94, 4 94, 3 91, 4 | 92, 4 94, 6 93, 2 92, 3 92, 3 90, 4 | 95, 4 93, 4 92, 3 90, 4 86, 4 82, 4 | 92, 4 90, 3 89, 4 85, 4 78, 4 73, 4 |
| 150 | 20 25 50 100 150 200 | 93, 0 95, 1 93, 4 93, 4 92, 4 92, 3 | 93, 4 93, 5 92, 3 92, 4 90, 4 86, 4 | 94, 4 91, 3 91, 3 88, 3 83, 4 79, 4 | 92, 4 92, 3 86, 4 79, 4 74, 4 70, 4 |
| 200 | 20 25 50 100 150 200 | 93, 0 92, 4 94, 2 92, 3 93, 3 91, 4 | 94, 4 95, 4 92, 3 90, 4 87, 4 84, 4 | 92, 3 91, 4 88, 4 84, 4 80, 4 75, 4 | 91, 3 90, 4 84, 4 76, 4 66, 4 58, 4 |
| 250 | 20 25 50 100 150 200 | 94, 4 96, 2 94, 4 93, 3 92, 3 90, 4 | 93, 2 93, 2 92, 3 89, 4 84, 4 82, 4 | 92, 3 92, 3 90, 3 83, 4 79, 4 71, 4 | 89, 4 88, 3 82, 4 74, 3 61, 4 56, 4 |
| 300 | 20 25 50 100 150 200 | 93, 3 94, 4 94, 4 93, 4 90, 4 90, 4 | 92, 3 92, 2 90, 3 87, 4 84, 4 80, 4 | 91, 3 90, 3 86, 3 81, 4 76, 4 67, 4 | 86, 4 88, 3 82, 4 67, 4 54, 4 46, 4 |

**Supplementary Table 4**. The 95% confidence interval coverage and bias of selected clinical record review (CRR) scenarios’ estimated harm rates. Coverage estimates that are significantly different from 95% are underlined. Scenarios vary by numbers of practices reviewing records, number of records reviewed in each practice and real harm rates (rHR)*. The median rate ratios (MRR) between patients and practices are 2 and 1.2 respectively. The results are from the beginning of the simulated 12-month period.

| Practices (n) | Records reviewed per practice (n) | 95% CI coverage (%), bias (%) | | | |
| --- | --- | --- | --- | --- | --- |
|  |  | rHR=2 | rHR=5 | rHR=10 | rHR=20 |
| 1 | 20 25 50 100 150 200 | - - - - - - | - - - - - 86, 20 | - - - 86, 20 85, 33 82, 20 | - - 84, 20 85, 20 75, 33 77, 30 |
| 10 | 20 25 50 100 150 200 | - - 87, 20 86, 40 80, 33 81, 30 | 89, 20 87, 28 87, 28 79, 28 70, 33 58, 32 | 87, 20 86, 28 77, 28 60, 32 49, 33 38, 34 | 78, 30 75, 36 62, 32 40, 30 25, 33 18, 32 |
| 20 | 20 25 50 100 150 200 | - 90, 20 87, 20 82, 30 73, 33 66, 30 | 88, 20 87, 28 78, 28 59, 32 48, 33 36, 32 | 83, 30 77, 28 62, 32 39, 32 27, 32 16, 32 | 67, 30 59, 32 40, 32 16, 33 7, 33 3, 32 |
| 50 | 20 25 50 100 150 200 | 88, 40 84, 28 75, 36 60, 32 51, 31 40, 32 | 80, 28 75, 28 59, 31 33, 33 15, 32 9, 31 | 59, 36 56, 31 35, 31 6, 32 3, 32 0, 32 | 37, 32 31, 33 9, 33 0, 33 0, 33 0, 32 |
| 100 | 20 25 50 100 150 200 | 82, 30 78, 28 61, 32 40, 32 25, 32 14, 32 | 60, 32 58, 31 32, 33 7, 32 3, 32 1, 32 | 36, 32 36, 31 7, 32 1, 32 0, 33 0, 33 | 12, 33 7, 32 0, 32 0, 33 0, 33 0, 32 |
| 150 | 20 25 50 100 150 200 | 72, 33 71, 33 49, 33 24, 33 9, 32 4, 33 | 48, 33 42, 32 16, 31 2, 33 0, 32 0, 32 | 27, 32 16, 32 2, 33 0, 33 0, 33 0, 33 | 5, 33 2, 32 0, 33 0, 33 0, 33 0, 33 |
| 200 | 20 25 50 100 150 200 | 67, 30 62, 32 37, 34 11, 33 4, 32 0, 33 | 38, 32 29, 33 7, 33 0, 33 0, 33 0, 33 | 14, 32 6, 32 0, 33 0, 32 0, 32 0, 33 | 2, 33 1, 32 0, 32 0, 32 0, 33 0, 33 |
| 250 | 20 25 50 100 150 200 | 60, 32 58, 31 34, 31 8, 32 2, 33 0, 33 | 32, 33 19, 33 3, 32 0, 32 0, 33 0, 33 | 7, 34 3, 31 0, 32 0, 33 0, 33 0, 33 | 0, 33 0, 32 0, 33 0, 32 0, 32 0, 33 |
| 300 | 20 25 50 100 150 200 | 59, 30 49, 33 23, 33 4, 33 1, 32 0, 33 | 25, 32 14, 32 2, 33 0, 33 0, 33 0, 33 | 5, 32 1, 33 0, 32 0, 33 0, 33 0, 33 | 0, 33 0, 33 0, 33 0, 33 0, 33 0, 32 |
